# Response and Lessons Learnt Managing the COVID-19 Crisis by School of Medicine, National University of Singapore

**DOI:** 10.15694/mep.2020.000092.1

**Published:** 2020-05-06

**Authors:** Dujeepa D. Samarasekera, Denise Li Meng Goh, Su Ping Yeo, Nicola Siew Pei Ngiam, Marion M. Aw, Mei Mei Lim, Suresh Pillai, Shuh Shing Lee, Malcolm Mahadevan, Alfred Kow, Yap Seng Chong, Tang Ching Lau

**Affiliations:** 1 Yong Loo Lin School of Medicine

**Keywords:** COVID-19, undergraduate medical education, pandemic preparedness, institutes of higher learning, disaster management

## Abstract

This article was migrated. The article was marked as recommended.

**Background:** Healthcare professionals are playing an important role in the recent COVID-19 outbreak. It is crucial that the health systems maintain their ability to train students and residents during this time. However, there is a paucity of literature on the measures taken by higher education institutions to ensure academic continuity. The aim of this article is to share the systematic measures that were taken during the COVID-19 pandemic by Yong Loo Lin School of Medicine, National University of Singapore.

**Methods:** We discussed our multi-faceted approach to protect students, staff and patients/ standardized patients during the COVID-19 outbreak that occurred during a pivotal time in the school’s academic calendar.

**Results:** Our approach to ensuring academic continuity and quality were based on best practices in the following areas: 1) A coordinated leadership and management process 2) Prioritising safety for all stakeholders 3) Dissemination of information amongst the stakeholders in a transparent and efficient way, and 4) Maintaining the rigour and quality of training.

**Conclusion:** The initiatives were implemented as we leveraged on the available infrastructure and the collective team efforts of all involved. Further research will be done to evaluate the usefulness of these measures. We hope that this article would be a useful reference for other schools as they evaluate their pandemic preparedness in the event that the COVID-19 outbreak affects their country or similar crisis event in the future.

## Introduction

The world has endured several waves of pandemic outbreaks. The earliest systematically documented episode was around 500 years ago, when the pandemic inﬂuenza was first recognised ( Morens, Taubenberger and Folkers, 2010). Following which, the 1918 influenza pandemic had the greatest impact. Recent years have seen the Severe Acute Respiratory Syndrome (SARS) pandemic in 2003, swine H1N1 virus in 2009, and in 2019- the COVID-19 outbreak.

During these times, healthcare professionals are at the frontline, providing critical services to the affected patients and the community. It is thus important to ensure that medical students and residents in training are equally equipped with the relevant skills and knowledge to react should the need arise. There is literature describing the development of pandemic preparedness modules to equip undergraduate students with essential skills ( Carney *et al.*, 2011; Walsh, 2019). Another important consideration is the non-interruption of training of medical students and residents. It is important to ensure continuity of learning as well as a continuous supply of doctors and specialists to the health system.

The curricula and teaching-learning activities in medical schools differ vastly from other faculties. Assessments are not conducted solely in examination halls nor are they solely focused on written components. Rather, clinical or patient-based practical elements are used. Instead of a modular program, medical schools usually adopt an integrated curriculum where faculty from different disciplines are involved in teaching. Moreover, the learning activities are not restricted to classrooms or laboratories within the campus. They are also sited in healthcare institutions.

Existing literature on the measures taken by healthcare training institutions to ensure academic continuity is sparse. Furthermore, the emphasis is usually on areas such as school closure, maintaining good hygiene habits and utilizing technology for e-learning. Recent articles include one by Park *et al.* from the Sungkyunkwan University School of Medicine, which detailed about Committee formation, rescheduling of break and timetable and running clerkships at another institution ( Park *et al*., 2016). However, the authors also pointed out that their measures were not as comprehensive in covering all aspects ( Park *et al*., 2016). Patil & Yan also previously shared about making PowerPoint ﬁles with the lecturer’s voice available online, the postponement of the Year 3 examination, and using telephone conference for an external examiner to participate in distinction viva-voce examinations ( Patil and Yan, 2003)

It is often when we experience a pandemic that we learn the most about the contingency measures required to mitigate the impact of dangerous pandemics. The aim of this paper is to share with the wider educational community, systematic and holistic measures put in place by the Yong Loo Lin School of Medicine, National University of Singapore (NUS Medicine) during the COVID-19 pandemic. We describe a multi-faceted approach to protect students, staff and patients/ standardized patients when COVID-19 appeared during a pivotal time in the school’s calendar viz high stake examinations, admission of new students and the electives period. We hope that the initiatives described would be useful considerations for others if faced with a similar situation.

## COVID 19 Pandemic

The COVID-19 outbreak began at the end of December 2019 when it was announced that clusters of people were infected with pneumonia in Wuhan, many of whom had been to the local seafood wholesale market ( Lum and Tambyah, 2020). Sequencing analysis revealed that the disease was caused by a novel coronavirus named SARS CoV-2 that has an extraordinarily high (96%) identity and relation to a SARS-like coronavirus found in bats ( Zhou *et al*., 2020). In a matter of weeks, facilitated by national and international travel, massive transmission between people occurred ( Heymann and Shindo, 2020). By 27 January 2020, the human-to-human transmission had been confirmed not just in Wuhan city, but also in the rest of China and in other countries. Singapore, a key centre in Asia for trade and tourism, reported its first imported case of COVID-19 on 23 January 2020 and local transmission on 4 February 2020 (( Lum and Tambyah, 2020). As of 23 February 2020, the number of infected people in Singapore increased to 89. On the international front, the outbreak was detected in 28 countries, infecting more than 70,000 and killing at least 2,467 ( New York Times, 2020).

### Background to NUS Medicine

NUS Medicine takes in approximately 300 students each year into its five-year undergraduate program. Students are taught the basic medical sciences in their first two years before going on to clinical placements in year three. At the start of our 115-year history, our medical curriculum was largely subject-based and was modelled after the British medical education until 1997 ( Samarasekera *et al*., 2015). In recent years, refinements and rationalization were made to the curriculum and subjects were integrated for better learning. For instance, there was an emphasis placed on clinical relevance during basic sciences lessons and the use of simulation-based learning environments ( Samarasekera *et al*., 2015). New teaching approaches such as collaborative learning cases were introduced. In clinical settings, students were embedded in healthcare teams to allow them to work in real clinical settings ( Jacobs and Samarasekera, 2012). To develop a holistic physician, longitudinal tracks such as “Health Ethics, Law and Professionalism” (HELP) and “Medicine and Society” were also included in the curriculum ( Samarasekera *et al*., 2015).

## Methods

### Ensuring academic continuity and quality

The mitigation processes and continuity were guided by the best practices on pandemic preparedness (World Health Organisation, 2020) and experiences with previous pandemics such as SARS in 2003. Our approach to ensuring academic continuity and quality were based on best practices in the following areas:

1) A coordinated leadership and management process

2) Prioritising safety for all stakeholders

3) Dissemination of information amongst the stakeholders in a transparent and efficient way and

4) Maintaining the rigour and quality of training.

## Results

• ***A Centralized System of Management***

The EduTeam Contingency Workgroup (ETCWG) was swiftly set up to deal with the outbreak. Its task included developing a set of precautionary measures to ensure the well-being of students and staff, implementing a business continuity plan, as well as implementing policies that came from other committees and agencies. The latter included committees from the University (e.g. occupational health and safety), the Academic Health Centre (the National University Health System (NUHS)), the Ministry of Health and the Ministry of Education.

The ETCWG was also responsible for the proper dissemination of information to all department heads, faculty, administration, without missing information or misinformation. This rigour was also applied to the information sent out to the students, their parents or guardians. A one-stop direct communication link was made available to all stakeholders to clarify the initiatives or to share any suggestions to improve the situation. This helped build the trust and confidence in the school and the disseminated information.

• ***Prioritising safety for all stakeholders***

The safety of the staff and students was paramount, and a number of measures were put in place to safeguard the school community against COVID-19 by focusing on early detection and reducing the risk of widespread transmission.

### Log of Visitors, Leave of Absence, Stay at Home Notice, Quarantine

At the end of January 2020, the University mandated that all visitors, students and staff declare online their official and personal travel plans for the period between January and March 2020. This was to facilitate contact tracing if the latter was required. Students and staff were also advised to defer their official travel to mainland China with immediate effect and encouraged to defer their personal trips to mainland China.

The University required staff and students to report if they were under Stay at home notice or under quarantine. Teaching, research and administrative staff returning from mainland China were required to be on a 14-day Leave of Absence (LOA) and work from home. Measures were put in place to support these individuals through this isolating period. This included reassuring students that they would have special considerations for any summative assessments, that teaching faculty will support them academically and minimize disruption to their studies. The University provided access to mental health support services to students who felt stressed etc. Food and necessities were also provided where required. Staff and students who did not comply these measures were handled accordingly under the protocol such as the NUS Code of Conduct and NUS student disciplinary procedures.

### Temperature Taking and Monitoring

Additional precautionary measures were put in place when the Ministry of Health declared on 7 February 2020 that the Disease Outbreak Response System Condition (DORSCON) level has been elevated to Orange. The Ministry of Health website https://www.gov.sg/article/what-do-the-different-dorscon-levels-mean gives an overview of the different DORSCON levels.

All students and staff were required to take their temperature twice daily with the first before coming to campus and to declare them online. These logs were monitored so as to enable early detection of clusters of fever, which may signify an early outbreak of COVID-19. All visitors to the buildings and offices at the University campus would need to undergo temperature screening at screening stations located within the school.

### Split Team Work Arrangement

The Business Continuity & Crisis Management Committee (BCCMC), led by a Coordinating Vice-Dean for COVID-19 measures and a Unit Incident Commander for NUS Medicine, assisted the Dean to operationalize the split teamwork arrangement. This was to ensure continued work productivity and to preserve the functional integrity of each team in the event a member of the team got infected.

Each department was split into Team A and Team B. These teams were segregated by location, with the critical functions represented in both teams. Staff from the two teams were advised to practice segregation during work and social times e.g. no face to face meeting (replaced by e-meetings; no meeting during mealtimes). Departments were instructed to trial this arrangement from 21 February to 26 February 2020 and to rectify any potential issues during the trial. Departments then entered the split team mode immediately after the trial period. However, this was further changed in March to allow only essential staff to report to work and other staff members to work from home. The arrnagements are further described in the Ministry of Manpower website https://www.mom.gov.sg/covid-19/general-advisory-for-workplace-measures

### Cross-healthcare institution movement

NUS Medicine staff and students were advised to avoid cross-healthcare institution movement. Thus, they were to refrain from going into the other entities within NUHS or other medical institutions unless they were working there. In addition, healthcare professionals working in the clinical settings were not allowed to enter other medical institutions located elsewhere even if the institutions belonged to the same cluster.

### Cleanliness

The school had also stepped up the frequency of cleaning and disinfection of areas with high human traffic on campus. All frontline staff donned personal safety equipment. NUS Medicine Communications also regularly sent out information on good hygiene practice.

• ***Transparency and consistency in information dissemination***

WHO had previously published the key takeaway lessons from SARS, and this included the importance of swift and transparent reporting, timely global alerts, and the use of a reliable press and electronic communications (World Health Organisation, 2003). While these are for the national level, we adopted the same principles in our school.

There was a large volume of information online, especially on social media, and some of the content was not very accurate. One way to circumvent this was for the school to proactively provide transparent and trusted information. This was done at multiple fronts, and to students and staff. The school sent a daily update on the number of students and staff who were under the Leave of Absence, together with other information such as recommendations by the Ministry of Health. The school also created the COVID-10 chronicles. This cartoon-based 1-page information helped address commonly asked questions and circulating myths ( https://nusmedicine.nus.edu.sg/medias/news-info/2233-the-covid-19-chronicles). Additionally, these comic strips reinforced the key messages visually and with a splash of humour. These created talking points and helped provide some stress relief during these tense times.

Through emails from the NUHS Chief Executive and Group Central Medical Board, the community was also updated whenever there was an infected case within the healthcare institution. Similarly, the University President and Provost also notified the students and staff and provide details on infected cases within the campus. While providing this information, these entities were mindful to protect the personal data of the infected individuals. The organizations also provided information about the decontamination and containment measures put in place. All these contributed to building trust and confidence.

• ***Maintaining the rigour and quality of training***

The occurrence of COVID-19 came at a pivotal time in the school’s calendar. Besides disrupting student learning and clinical postings, other activities such as high stakes examinations and the admission exercise had to be looked into.

### Curriculum

All co-curricular non-essential events and activities in the school involving more than 50 participants were either cancelled or deferred until further notice (e.g. community service projects, overseas humanitarian trips).

For the pre-clinical phases that consisted of primarily lectures and tutorials, e-learning was implemented for classes with more than 50 students. Face-to-face classes were allowed only for class sizes of 50 and below. For such face-to-face classes, besides taking attendance, instructors also took pictures of all attendees present, in order to document where each student was sitting. These were to facilitate contact tracing purposes if the need arose. Hospital attachments for these pre-clinical students were replaced by procedural skills, simulation training using task trainers, as well as simulated encounters with SPs in the Simulation Centre.

Students in their clinical rotations in the various healthcare institutions had, their clinical rotations stopped. In addition, their 2-week break was brought forward, and their examinations deferred to a later date. This allowed the academic staff to re-organize the clinical training to alternative means (e.g. case-based learning, task trainers, simulation).

This move from clinical to technology-enabled teaching could be accomplished as the school had already been using an e-learning platform to complement learning. Hence, students and a substantial number of staff were familiar with such tools. In addition, these resources were well documented so staff could easily learn how to use this if they had not done so before. For example, the Education Technology Team at NUS Medicine has previously documented a manual with step-by-step instructions on using e-learning tools such as Entrada. The content of the manual includes:

• Uploading past webcast link and lecture slides
• Creating online quizzes
• Recording narrated lecture slides
• Setting up and conducting online meetings

In addition, the University’s Centre for Development of Teaching and Learning had a wiki that provided resources on many IT tools ( https://wiki.nus.edu.sg/display/cit/Wiki.nus). They also quickly organized a series of webinars and some classroom sessions to train all the interested teaching staff.

### Admissions

The University’s Open House had always been held on campus with many meet the people sessions. In view of the pandemic, the University switched to an e-open house. Instead of the usual one to a two-day event over a weekend, the planned e-open house ran over 9 consecutive days and comprised of live webinars, pre-recorded faculty talks, online chats and virtual tours showcasing student life at the campus. Instead of having potential candidates watch live simulation sessions at the Open House, videos of the various teaching activities and simulation sessions were included on-line for viewing.

The usual admission exercise to NUS Medicine involves assessing candidates via a 5-station Focused Skills Assessment (FSA) as well as other evaluation tools such as interviews, a portfolio station and a written (MCQ) Situational Judgment Test (SJT). The FSA included 2 stations where candidates’ interactions with SPs were observed, a task-based problem-solving activity station, as well as a team station where 4-5 candidates worked together on a task. The aims of the multi-station FSA were to assess candidates for non-cognitive attributes such as communication skills, empathy, integrity, teamwork and resilience, as well as their prioritization and problem-solving skills.

During the COVID-19 situation, we modified the admissions exercise by reducing the FSA to a 2-station exercise, held on campus via an on-line Zoom platform. The 2 stations were the portfolio station (similar to the usual admissions exercise) and a customized “new” scenario-based station. The latter comprises scenarios similar to that asked in the SJTs, except that it would be the assessor verbally discussing the candidate’s responses and thinking behind those responses. The discussions were done via video, with the assessor and candidate being in separate rooms or at their homes. Having a “new” station for the admissions exercise meant that additional training sessions for the assessors would need to be arranged.

Reducing the FSA from 5 stations to 2 stations also meant a reduced number of assessors required. As there was a nationwide ban on healthcare professionals from different institutions meeting or mingling, this logistic constraint was also a consideration taken into the decision making, as it enabled NUS Medicine to hold the admission exercise tapping on the only faculty based on its NUHS campus.

### Electives

A new structure had to be put in place for the Year 4 electives, where clinical placements were replaced with non-clinical elective options. These new options included two initial weeks of e-lectures to frontload the key core content. Students would then choose to work on a project. This project could be one already offered as an existing non-clinical elective project, or one that the students chose to work on as a group. The latter should focus on addressing the gaps in the curriculum. Students would be required to do a preliminary presentation of their findings in Week 6 of the program, and to receive feedback. If the clinical electives were not resumed by Week 6, the students would then continue to work on the project and do a final presentation in Week 10.

### Exams

The COVID-19 outbreak coincided with the start of our peak exam season, with 4 out of the 5 years having their high stakes end of year examinations slated to occur between February to May 2020. The school made several modifications to the assessment format. The principles guiding the modifications were 1) infection control measures; 2) being mindful of how the students had been trained and how they had been preparing for the examinations; 3) reducing the fall out due to fear of contracting COVID-19.

Our theory exams were originally slated to run en masse (i.e. 300 students at a time) in a single location. This posed increased risks of transmission of infection and also increased risk to the nation in the event of having to quarantine those exposed. To reduce these risks, the theory exams were decentralized and conducted simultaneously in many different sites, with each site hosting only 50 students. Temperature screening and contact screening were conducted prior to entry into the examination. Proctoring was done at each site; this was possible as we had enough staff who knew how to run the exams and troubleshoot issues on their own. Exam briefing was done via the use of centrally produced e-materials. One chief invigilator proctored centrally via electronic contact. The school’s technology team provided technical support centrally. An additional benefit of using E-exam was the reduction of the risk of the hard copy exam papers being a fomite in the transmission of COVID-19, and the ability for examiners to mark remotely via electronic means.

The clinical exams in the school traditionally involved multiple days, multiple stations, multiple real patients, multiple standardized patients, multiple examiners, and multiple staff, with many hailing from different healthcare institutions (i.e. a mass gathering). For our graduating class alone, each of our ~300 students would need to be assessed in 21 stations spread over 5 days. These mounted to 6,300 different touchpoints (excluding registration etc.).

To reduce the risk of infection, the risk of transmission and the risk of quarantine, the following measures were put in place. The exams were held in a non-clinical institution. The students would follow the same grouping for their clinical exams as they did for their theory exams. These groups reported at different times and were held in different rooms. Temperature screening and contact screening were conducted prior to entry into the examination. Surgical masks were provided to all. Seventy per cent alcohol-based hand sanitizers were made widely available for use. Large briefings were decentralized to small group briefings. Where possible, e-briefings were done prior to the examination.

For examiners and staff, similar infection control measures were put in place. We also reduced the number of examiners involved by encouraging examiners to examine the whole day instead of half a day (with the provision of adequate breaks and rests in between sessions). In addition, we reduced the number of healthcare institutions involved on each day and also cohorted examiners by their institutions. Meals were provided as packed lunches to be eaten alone, as opposed to previous years where there was one central buffet for all to gather to eat together.

One of the central tenets in the making these changes was to reassure all participants their own safety and to avoid no shows of examiners, standardized patients, administrative staff and/or patients. Such no show would jeopardize the ability to run a valid exam. This concern was managed by sharing the infection control measures put in place and also allowing for questions and clarifications. We also made the decision early not to use any real patients. This was based on our experience during the SARS period where the no show rate in-clinic increased substantially due to patients being afraid to go near a health care institution. By not using any real patients, it meant that we had to change 9 out of the 21 stations to SPs, simulators and task trainers. A new exam blueprint was developed. Cases were then written up, reviewed and validated for the stations. Examiner training and standardization was also carried out. Measures were also put in place to look at the post hoc exam statistics to assess if there were any detrimental effect of these changes.

We also had to manage the concerns of the graduating class as this occurred less than a month prior to the final examinations. We conducted a detailed interactive briefing via live streaming to the entire class. This was also followed up by the provision of more information via emails and our learning management system. This final exit exam is high-stakes, as it determines whether the student graduates or not. There was a concern that students, despite being unwell, would be tempted to turn up for the examination. As such, students were reassured that there would be a special makeup exam in the event that they were unwell, quarantined or infected. Extra self-directed learning sessions using simulators and task-trainers for final year students preparing for OSCE exams were organized by the Centre for Healthcare Simulation. The students were also told very early on in the outbreak to do social distancing and to practice good hygiene so as to reduce the chances of infection and quarantine.

## Discussion

### Strengths

#### Technology

As the school has planned for the contingency measures and invested in technology, NUS Medicine was able to tap on the available online resources and to do so quickly. This minimized the impact of the COVID-19 outbreak on key activities such as learning and exams. Besides helping learning activities, the use of technology had also allowed for key discussions, decision-making and timely communication to the relevant stakeholders.

#### Rapid Mobilization of SPs

SPs were mobilized very quickly to meet the needs of the curriculum and examinations. We were able to do this because we had a reliable and up to date database of SPs who work with the school. SPs with special skills, like the physical examination teaching associates, were able to assist with the teaching of the physical examination that traditionally were taught with the presence of real patients.

#### Collective NUSMed Team Effort with Strong Leadership

A participative approach was adopted in the formulation of the contingency learning event schedules for students, forging partnerships between students and academic faculty. This was necessary since the students were the primary stakeholders and who were also the ones affected the most.

The school has always focused on continuous improvement of the educational and working environment via input from the staff and students. This likely translated to a more resilient student and staff community who were able to put in the extra effort and to rapidly adapt to the measures set in place. For instance, the official announcement on the split teamwork arrangement was only sent a day before the trial was supposed to begin. Despite the short notice, the majority of the school were able to respond swiftly and find alternative venues within the campus to work during the trial period. This was not an easy feat as the school has more than 20 any of the senior members implementing these changes had lived through SARS. They could draw on their past experience to add value to the plans. Their calmness when leading the teams helped keep the anxiety in check.

Finally, decision making and implementation processes were efficient, primed by the situational leadership style adopted at University, school, divisional and committee levels. The fact that the final MBSS contingency plan was shaped up in a timely manner (<2 weeks) provided assurance to stakeholders about the school’s robust assessment system and operations, without compromise on the principle of validity and reliability.

### Limitations

The implemented measures were not without limitations. In the case of assessment, the modifications may likely reduce the validity of the assessment method. For the admissions exercise, there is a concern whether the 2 stations can have the same robustness and reliability as 5 stations. For learning, the switch to online lessons limited the face-to-face interactions and could change the dynamics between the students and tutors. We hope that by encouraging lecturers to use online platforms to engage the students in discussion, the tutor-student interaction would still be of value in learning. Lastly, the new elective format had reduced the extra clinical and/or overseas exposures which many students in previous years have found valuable in their holistic development.

While we believe that we have done the necessary to ensure academic continuity and quality during this period, we are planning to conduct impact studies on student learning experience and performance to further evaluate our methods.

## Conclusion

In summary, we shared in great depth the contingency plans put in place by NUS Medicine, covering a wide span of program-related aspects from the curriculum, admission, electives to examinations, in addition to safety measures for all stakeholders- students, faculty and staff. These implementations were based on the 4 areas we described which enabled the key stakeholders to develop trust, confidence and conform to the stipulated guidelines which led to workplace safety. We hope that these would be a useful reference for other schools as they evaluate their pandemic preparedness during and after the COVID-19 outbreak.

## Take Home Messages

• The recent COVID-19 pandemic has interrupted the training of medical students.
• We shared the systematic and holistic measures we took to protect students, staff and patients/ standardized patients while ensuring academic continuity and quality.
• Our approach was based on best practices in the following areas: 1) A coordinated leadership and management process 2) Prioritising safety for all stakeholders 3) Dissemination of information amongst the stakeholders in a transparent and efficient way, and 4) Maintaining the rigor and quality of training.
• The measures were implemented without much difficulty as we tapped on the available technological infrastructure and the collective team efforts of all involved.

## Notes On Contributors

**Dujeepa D. Samarasekera** is the Director of the Centre for Medical Education, Yong Loo Lin School of Medicine, National University of Singapore, Singapore and Senior Consultant at Ministry of Health, Singapore. ORCID iD: https://orcid.org/0000-0002-6916-6741

**Denise Li Meng Goh** is an Associate Professor and Chair of the Faculty Assessment Committee at the Yong Loo Lin School of Medicine, National University of Singapore, Singapore; Senior Consultant at the Department of Paediatrics, National University Hospital, Singapore

**Su Ping Yeo** is a Senior Executive at the Centre for Medical Education, Yong Loo Lin School of Medicine, National University of Singapore, Singapore.

**Nicola Siew Pei Ngiam** is the Head of the Standardized Patients Programme at the Yong Loo Lin School of Medicine, National University of Singapore, Singapore; Paediatrician at the Department of Paediatrics, National University Hospital, Singapore.

**Marion M. Aw** is the Assistant Dean (Education) at the Yong Loo Lin School of Medicine, National University of Singapore; Associate Professor and Consultant Paediatrician at the Department of Paediatrics, National University Hospital, Singapore.

**Mei Mei Lim** is a Deputy Director at the Dean’s Office (Education), Yong Loo Lin School of Medicine, National University of Singapore, Singapore.

**Suresh Pillai** is the Director of the Centre for Healthcare Simulation, Yong Loo Lin School of Medicine, National University of Singapore, Singapore; Senior Consultant at the Emergency Medicine Department, National University Hospital, Singapore

**Shuh Shing Lee** is a Medical Educationalist at the Centre for Medical Education, Yong Loo Lin School of Medicine, National University of Singapore, Singapore.

**Malcolm Mahadevan** is the Assistant Dean (Education) at the Yong Loo Lin School of Medicine, National University of Singapore; Head and Senior Consultant of Emergency Medicine Department at the National University Hospital, Singapore.

**Alfred Kow** is the Assistant Dean (Education) at the Yong Loo Lin School of Medicine, National University of Singapore; Senior Consultant Surgeon at the University Surgical Cluster, National University Hospital, Singapore.

**Yap Seng Chong** is the Dean of Yong Loo Lin School of Medicine, National University of Singapore, Singapore.

**Tang Ching Lau** is Vice-Dean (Education) of Yong Loo Lin School of Medicine, National University of Singapore and a Senior Consultant Rheumatologist in the University Medicine Cluster in the National University Hospital, Singapore.
