# Supplementary material for: Response and Lessons Learnt Managing the COVID-19 Crisis by School of Medicine, National University of Singapore
Source: MedEdPublish (2016). 2020 May 6;9:92. [Version 1] doi: 10.15694/mep.2020.000092.1 (PMC10697446; doi:10.15694/mep.2020.000092.1)
Supplement: Peer review history

# Reviewer report: Reviewer response for version 1

**DOI:** 10.21956/mep.20056.r30759

**Published:** 2020-05-22

**Authors:** Hui Meng Er

**Affiliations:** 1 International Medical University

This review has been migrated. The reviewer awarded 5 stars out of 5 This is an excellent article that describes the concerted efforts of all departments of the university in addressing the impact of COVID-19 outbreak on education to ensure academic continuity and quality. It highlights the importance of engaging the stakeholders in formulating and implementing the contingency plans during COVID-19 pandemic or other similar situations. It is definitely a useful reference for crisis management in education settings. It is not unexpected that varying extent of challenges may be encountered during the implementation. Some discussion on the examples and strategies to manage/overcome them would be valuable for the readers, for example, in dealing with issues related to the acceptability of the plans and possibly stakeholders’ resistance. The impact studies on student learning experience and performance as suggested by the authors will provide further evidences on the effectiveness of the implemented measures.

Reviewer Expertise:

NA

No decision status is available

# Reviewer report: Reviewer response for version 1

**DOI:** 10.21956/mep.20056.r30756

**Published:** 2020-05-19

**Authors:** Mohamed Al-Eraky

**Affiliations:** 1 University of Dammam

This review has been migrated. The reviewer awarded 5 stars out of 5 This is a timely study that proposed a comprehensive approach to manage the COVID-19 crisis in academic institutions. I may suggest two points. First, to further elaborate on the Methodology section to help colleagues in other contexts. Second, to plan a follow up article after few months to report the effectiveness of measures taken. This has been already proposed by the authors that further research will be done to evaluate the usefulness of these measures. Thank you and good luck.

Reviewer Expertise:

NA

No decision status is available

# Reviewer report: Reviewer response for version 1

**DOI:** 10.21956/mep.20056.r30758

**Published:** 2020-05-10

**Authors:** P Ravi Shankar

**Affiliations:** 1 American International Medical University

This review has been migrated. The reviewer awarded 4 stars out of 5 This article provides a comprehensive review of measures taken at a medical university in Singapore during the COVID-19 pandemic. The authors provide a very brief history of pandemics. I am not sure about whether there had been operational medical schools during the plague pandemics and would have been interested in whether they were able to function during pandemics. Among the things which supported the school’s response to the pandemic were their investment in an online educational system and their previous experience of dealing with the SARS outbreak. The authors provide a background to recent curricular changes at NUS medicine. The current outbreak happened during a critical period in the academic calendar. The authors mention the limitations of their measures and plan to do impact studies in the future. The paper is well-written and provides an overview which will be useful to other medical schools. The changes in the admissions process and in the open houses were of special interest to me. The impact of these changes which occurred quickly due to the rapid evolution of the pandemic on students, faculty members, other staff, examiners and standardized patients will also be of interest.

Reviewer Expertise:

NA

No decision status is available

# Reviewer report: Reviewer response for version 1

**DOI:** 10.21956/mep.20056.r30757

**Published:** 2020-05-07

**Authors:** Balakrishnan Ashokka

**Affiliations:** 1 National University Health System (NUHS)

This review has been migrated. The reviewer awarded 5 stars out of 5 ABOUT THE ARTICLE: • The authors provide a comprehensive work, detailing the actual work done in coping with high stake final professional exams in an academic teaching hospital that had previous experience with SARS.• The coordinated responses, the decision to eliminate real patients and ramping up SP supplementing that within a span of two weeks is to be applauded.• While changes were made for this transition to occur, all stake holders were supported in the process with Edutech support for both faculty and students while broad measures of safety, hygiene and social distancing were implemented. WHAT VALUE IT ADDS:• For the world of simulation this is a home run!! ..When finally assessments that were a concern when simulation was used for high stakes, has eventually proven to be considerable help.. While this doesn’t replace the issues and inadequacies of simulation, it proves to be a valuable high-stake assessment tool …• This serves like a blue a print of planning a systematic response and implementation process for medical school that have to cope with conducting assessments while keeping everyone safe• The lockdown phase is coming to an end, and businesses, hospitals and eventually universities have to resume “business as usual” and this would be an benchmark article that could suggest a proven example for all academic universities and how to plan these activities until the pandemic fizzles off.• Resource limited settings with higher student volumes (0f more than 200 students) that cannot afford indefinite postponements and have the pressure of academic calendar can consider the suggestions for segregated teaching and learning and virtual examiners. SCOPE FOR FURTHER WORK AND FUTURE DIRECTIONS:• The education impact and outcomes of the modified and redesigned assessments must be ascertained. We look forward to the impact studies that the authors have quoted are under way• It will be useful to hear from the examiners: “what they felt were their real concerns when they have to now assess students virtually, or through simulated clinical encounters” and the student’s opinions on… “ what they felt were their anxieties when they had always had patient-based end of posting tests and now suddenly have to be assessed mostly through simulated patients and how they felt they were ready as interns having passed a modified final exam”I wish the authors congratulations on their commendable work and will look forward to end user feedback. Ashokka Balakrishnan MMBS MD FANZCA EDRA MHPE (Anaesthesiologist, Medical Educationalist and Simulation Program Director-Anaesthesia)

Reviewer Expertise:

NA

No decision status is available
